# Divulging the Impetus of Yoga on Cardiorespiratory Fitness and Its Persona in Alleviating Anxiety Experienced by Youth: A Cohort Interventional Study

**DOI:** 10.7759/cureus.38847

**Published:** 2023-05-10

**Authors:** Gaurav Mittal, Ruchi Kothari, Akshay Yadav, Pradeep Bokariya, Prashanth A

**Affiliations:** 1 Physiology, Mahatma Gandhi Institute of Medical Sciences, Wardha, IND; 2 Anaesthesiology, Lokmanya Tilak Municipal Medical College and General Hospital, Mumbai, IND; 3 Anatomy, Mahatma Gandhi Institute of Medical Sciences, Wardha, IND

**Keywords:** medical student training, psychiatry and mental health, spielberger’s scale, anxiety score, vo2 max, cardiorespiratory fitness, yoga research

## Abstract

Background: Globalization endangers youngsters worldwide with new standards and possibilities. Hereat of being exposed to greater demands and expectations, when it comes to performance review, their life may become more distressed. Yoga with revolutionary methods may assist youngsters in bettering their physical health regarding their maximal oxygen uptake, and also help manage their anxiety. This study ascertains the effect of yoga on youth's anxiety levels and cardio-respiratory fitness.

Methods: It was a longitudinal interventional study recruiting 99 medical students wherein VO_2_ max (maximal oxygen uptake) on the treadmill/ergometer exercise and anxiety scores through Spielberger's anxiety scale was assessed at baseline and evaluated after 6 months of a regular yogic regime. The VO_2_ max was recorded by the metabolic module of Labchart software (Bella Vista, New South Wales, Australia).

Findings: The VO_2 _max evaluated by incremental exercise to volitional fatigue was found to be 2.64 ± 0.49 L/min in males and 1.51 ± 0.44 L/min in females pre-yoga and 2.81 ± 0.52 L/min in males and 1.69 ± 0.47 L/min in females post yoga. The difference in the endline and baseline VO_2_ max values of yoga-performing males (t=6.595, p<0.001) and females (t = 2.478, p = 0.017) was found to be significantly higher than non-yoga performers. The METS value obtained in males was 11.96 and in females was 7.68 before yoga. Post-yoga values were 13.44 and 8.37, respectively. The difference in total anxiety scores post-intervention was 34.6 which was statistically significant (t= 4.959, p <0.001).

Conclusion: From the viewpoint of a physiologist, higher VO_2_ max in young adults links to better physical fitness which is the potential outcome of regular yogic practice. As a result of regular yogic practice, initial soaring anxiety levels of subjects culminated in a drastic observable reduction in anxiety, which helped inculcate a judicious acumen in youngsters.

## Introduction

This article was previously presented as an oral presentation at the 2022 Asia-Singapore Conference on Sports Sciences on December 6, 2022.

Youth are considered the blossoming buds of progression for any dwelling society, population, or even a nation as a whole. The fast-flourishing metamorphosing world of cut-throat competition is boggling with stress. It is being manifested as the most common problem among the modern generation. On entering into professional colleges, students find themselves in a new, challenging, and stressful environment. Particularly, it has been observed in medical students that they experience significant stress during their course [1-2]. Numerous factors that contribute to soaring levels of stress in medical students could be a highly competitive curriculum, intense academic competition, and excessive demands on coping abilities in physical, emotional, intellectual, financial, and social terms. 

As per literature available from the West [3-5], and from Asia [6] it has been documented that medical training is highly stressful, particularly for those who are in the cradle stages of their medical schooling. It is well known that stress modifies the autonomic nervous system's fineness, with sympathetic activity predominating in anxious temperament. Yogic praxis has benefitted in maintaining a physiological milieu pertaining to cardiovascular indices [7]. Yogic relaxation can moderate sympathetic preponderance [8-9]. By minimizing sympathetic activity, yoga asanas, and pranayama can tip the autonomic equilibrium in favor of relative parasympathetic control [9-11]. The objective manifestations of anxiety -- a racing heart, palpitations, sweating, elevated blood pressure, dry mouth, avoidance behavior, signs of restlessness, and heightened responsiveness reduce and eventually vanish.

Medical school comes in like a soft breeze after the post-entrance inertia of rest, holidays, and fun in the life of medical students who are no longer just medical aspirants. In no time this ‘soft’ breeze gets transformed into a storm and forsooth wreaks havoc on their mental and physical well-being. Here the age-old praxis comes to their rescue anon, which is none other than yogic practice. Yoga in today's so-called “sophisticated cosmos” is described as an antediluvian practice, especially among the youth who are at crossroads. In the age of mobile phones, beepers, and 24 x 7 browsing, the yogic practice seems a more pertinent way out. Yoga practitioners have asserted its effect on balancing emotional, physical, and spiritual health for decades, but only recently has there been a move to substantiate these claims through research [12]. 

A state of mental tranquility is achieved by the practice of yoga as revealed by an increase in alpha waves of electroencephalogram after yoga [13-14]. At the physical level, consistent practice of asanas and pranayama confers a proportionate, flexible, typically relaxed body with an ability to combat stress efficiently [15]. Recent research has made some assertions that yoga has revolutionary methods for assisting youngsters in bettering their physical health as measured by their VO₂ max, which could also impact how they manage their anxiety. According to MI [16], the maximal oxygen uptake, i.e. VO₂ max is the single best measure of cardiorespiratory efficiency and the gold standard of physical fitness for any individual. Keeping in mind the above-stated facts, it was thought pertinent to probe an answer to the arising question as to whether long-term yoga practice could prove to be a boon for the young generation of today's world with intense academic aspirations.

This study aimed at assessing the effect of a regular yogic regime on youth's degree of anxiety and cardio-respiratory fitness in terms of aerobic capacity as assessed in the Sports Physiology Laboratory.

## Materials and methods

Study design and setting

It was a cohort interventional study with pre- and post-design. The Strengthening the Reporting of Observational Studies in Epidemiology (STROBE) guidelines were used for reporting and preparing the manuscript. The study was carried out in the Sports Physiology Laboratory of a rural medical college in central India. It was undertaken where the parameters of cardiorespiratory fitness in subjects were evaluated first at baseline and later after completion of a yogic regime. During the first month, all the recruited subjects practiced yoga together under the leadership of the investigator and a trained yoga expert. Then they were advised an hour of daily yoga for a duration of 6 months, and the same subjects were re-evaluated. Similarly, baseline assessments of anxiety levels were made (pre-yoga). Subsequently, the anxiety scores were assessed following a month of yoga then there was a six-month (post-yoga) follow-up period during which the students engaged in independent praxis. We obtained signed written informed consent from all study participants. Prior approval from the Institutional Ethics Committee was ensured before the beginning of the study. 

Study population and selection criteria

Students pursuing Bachelor of Medicine, Bachelor of Surgery (MBBS) through a rural medical college were recruited for the study. Initially, students were explained about the study and how it can benefit them in the longer run. The students who volunteered and were willing to participate in the study were shortlisted. The research was then undertaken accordingly.

The sample size was estimated using OpenEpi 3.01 statistical software (Centers for Disease Control and Prevention - CDC, Atlanta) with the assumptions as the confidence level of 95%, alpha of 0.05, and power of study as 80%. The minimum sample size came out to be 84 according to the statistical software. During scrutinizing, 300 MBBS students were screened. As per the inclusion and the exclusion categorical imperative, only 99 students were considered for the study which was well over the calculated sample size. Considering a 10% shift of mindset of the students for not participating in the study, all 99 were recruited for the baseline assessments.

The MBBS students in the age range of 17-25 years who gave written informed consent were included in the study. Subjects should not be involved in heavy physical activity or sports for at least a year. They should not have any already ongoing yogic regime which might already have an effect on the baseline values.

Subjects suffering from any acute illness, recent surgery, endocrine disorders, cardiovascular disorders, COPD/asthma, chronic debilitating diseases such as cardiac arrhythmias, diabetes, persons receiving any drug that may affect the autonomic reflexes; not giving consent, and not willing to participate were excluded. 

**Figure 1:**
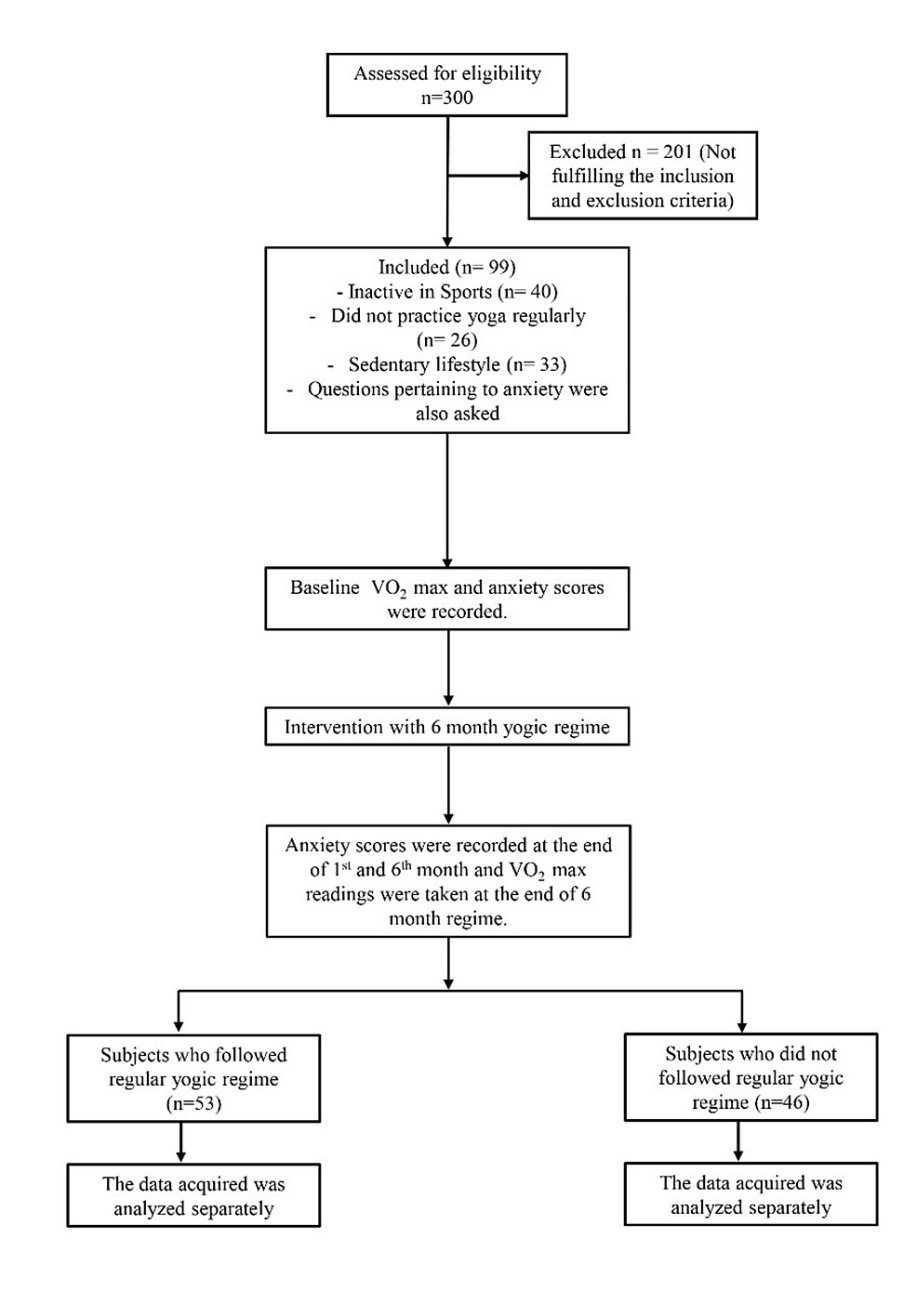
Flow diagram showing recruitment of participants. 'n' denotes number of participants

The flow diagram in Figure 1 shows the final recruitment of participants after fulfilling the selection procedure. 

While skeletonizing the subjects of the study it was found that 33 had a totally sedentary lifestyle while the other 66 were not involved in any kind of heavy physical pursuits which could have caused bias or might have confounded the outcomes. According to the final outcome as described above, the initial cohort was divided into two groups namely a yoga performer group and a non-yoga performer one. The yoga performer group comprised subjects who followed a regular 6-month independent yogic regime and the non-yoga performer group involved students who irregularly practiced yoga. Regularity was kept a check upon by the investigators by asking for regular reports about the same. The ones who reported yogic practice for more than 75% of the days during the 6-month independent praxis were considered regular practitioners. 

Data sources and measurement of variables

The following parameters were investigated pre- and post-yoga to determine the relationship between yogic practice and physical and mental fitness.

i) O₂ max - The level of oxygen consumption beyond which no further increase in oxygen consumption occurs with a further increase in the severity of exercise.

Procedure for VO2 max

After familiarization with the laboratory and procedures, the subjects performed an incremental ramp exercise test for volitional fatigue on a motorized treadmill/ergometer. Treadmill/ergometer speeds were predefined to increase incrementally from moderate to maximal effort. VO2 max was thus measured by this symptom-limited running exercise. A valid VO2 max was considered to have been attained when the following criteria were achieved:

a. Plateau or 'peaking over' in oxygen uptake / O₂ consumption (VO₂).

b. Achievement of maximum heart rate: 220 - Age

ii) METS - One metabolic equivalent (MET) is defined as the amount of oxygen consumed while sitting at rest and is equal to 3.5 mL O₂ per kg body weight times the minutes of exercise. The MET concept represents a simple, practical, and easily understood procedure for expressing the energy cost of physical activities.

Calculation of METS

Energy cost/expenditure (METS) is used as an indicator that the participants are nearing exhaustion and the limits of their cardio-respiratory system. METS values were recorded as per the maximal oxygen consumption output data.

The metabolic module of Labchart software was used to process the data and give the output readings. The Power lab 8/35 data acquisition system was used for the recording of VO2 max and deriving METS Values. Increasing workloads are used to reach exhaustion in the subject and determine a maximal level of oxygen consumption (VO2 max). A motorized treadmill (Aerofit AF 101, Nityasach Fitness Pvt Ltd, Mumbai, India) was used for the subjects to perform and reach maximal exercise levels.

Baseline clinical parameters - resting pulse, blood pressure, and resting respiratory rate were measured. The height and weight were recorded as per standard procedures. 

iii) Anxiety levels were measured into three broad categories namely:

a) Trait anxiety which is an enduring characteristic or pattern of behavior and refers to the more stable tendency to attend to, experience, and report negative emotions such as fears, worries, and anxiety across many situations. This is part of the personality dimension of neuroticism versus emotional stability.

b) State anxiety which implies that state is a temporary way of being (i.e., thinking, feeling, behaving, and relating)

c) Combined anxiety score

Tool for Assessment of Anxiety

Spielberger's anxiety scale [17], which is a standardized, validated, and widely used measure to determine the anxiety score of students, was employed. It includes a questionnaire called the ‘State-Trait Anxiety Inventory’ (STAI). A self-report assessment device, which includes two separate subscales containing 20 items each. It measures state and trait anxiety using a four-point Likert scale. Essential qualities evaluated were - feelings of apprehension, tension, nervousness, and worry.

Anonymous feedback was also taken at the end of the intervention to understand students’ experience of yoga using a Proforma in which 13 parameters were assessed. Students were asked to tick against the column which was most appropriate with regard to their experience for each of the parameters. The number of students who have chosen a particular grade is expressed as a percentage of students. A consensus measure was calculated for the items on the Likert scale using the method of Tastle et al. [18].

Statistical data analysis

Initially, Kobo Toolbox was used to collect anthropometric and historical data while screening the students. For quantitative data collection of variables like VO2 max, METS, and results of the anxiety scores according to the Likert scale, also Kobo toolbox was made use of. After that, R Software [19] (R Foundation for Statistical Computing, Vienna, Austria) was utilized for statistical analysis and preparation of the graphs. Certain scores of anxiety levels were found to deviate from normal distribution after their combined results were checked for normal distribution. Subsequently, the non-parametric Wilcoxon signed-rank test was performed, which is used to compare two averages. Mean and standard deviation, the difference in means of various parameters, correlation analysis using Pearson’s coefficient which was deemed significant depending upon the outcome of the p-value. If the p-value was < 0.05, it was considered to be significant. Paired t-test was employed for analyzing pre- and post-intervention scores of variables.

## Results

Participants' descriptive data

The mean age of yoga performers was 19.43 ± 2.62 years and 19.54 ± 2.59 years for the non-yoga performers. There was no statistically significant difference found between these (p-value = 0.76). Reasonably mean height and weight of both the groups were found to be on similar lines which proved the point that demographic parameters did not confound the readings. There were 25 males and 21 females in the non-yoga performing group of participants and 33 males and 21 females in the yoga performers. The VO2 Max readings were taken for both the groups after 6-month intervention while the anxiety scores were recorded only for the students who regularly performed yoga (n=54).

Main outcomes and results

Tables 1-2 below give a comprehensive insight into the maximal oxygen consumption data (VO2 max) readings of the yoga performers as well as non-yoga performers respectively. Pre- and post-yoga readings categorized as per sex and in two defined units, have been depicted. The METS value was found to be higher post-intervention which is 11.52 compared to the baseline value of 10.34 and this difference was found to be statistically significant (p<0.05).

**Table 1:** Maximal oxygen consumption data of yoga performers group.

Sex	Pre-yoga reading		Post-yoga reading	
	VO_2 _max (mL/kg/min)	VO_2_ max (L/min)	VO_2_ max (mL/kg/min)	VO_2_ max (L/min)
Males (n=33)	41.86 ± 6.16	2.64 ± 0.49	44.52 ± 6.21	2.81 ± 0.52
Females (n=20)	26.95 ± 4.94	1.51 ± 0.44	29.99 ± 4.94	1.69 ± 0.47

**Table 2:** Maximal oxygen consumption data of non-yoga performers group.

Sex	Pre-yoga reading		Post-yoga reading	
	VO_2 _max (mL/kg/min)	VO_2_ max (L/min)	VO_2_ max (mL/kg/min)	VO_2_ max (L/min)
Males (n=25)	27.95 ± 5.24	1.99 ± 0.50	28.29 ± 5.39	1.93 ± 0.48
Females (n=21)	17.08 ± 2.40	1.14 ± 0.28	18.33 ± 8.10	1.24 ± 0.67

Table 3 shows the difference in VO₂ max in mL/kg/min and VO₂ max in L/min and METS before and after intervention in both groups. For VO₂ max in mL/kg/min in the yoga performer group, the mean difference was found to be higher 2.8 compared to 0.15 in the non-intervention group and the difference was found to be statistically significant. Similarly, for METS and VO₂ max in L/min, before and after intervention value difference was found to be higher in the yoga performer group compared to the non-performer group, and the difference was found to be statistically significant (p-value = 0.0004).

**Table 3:** Difference in difference analysis of fitness parameters. METS, one metabolic equivalent

Parameters	Yoga performers group	Non-Yoga performers group
Difference in VO₂ max (mL/kg/min)	2.80	0.15
Difference in VO₂ max (L/min)	0.17	0.02
Difference in METS value	1.18	0.02

**Figure 2:**
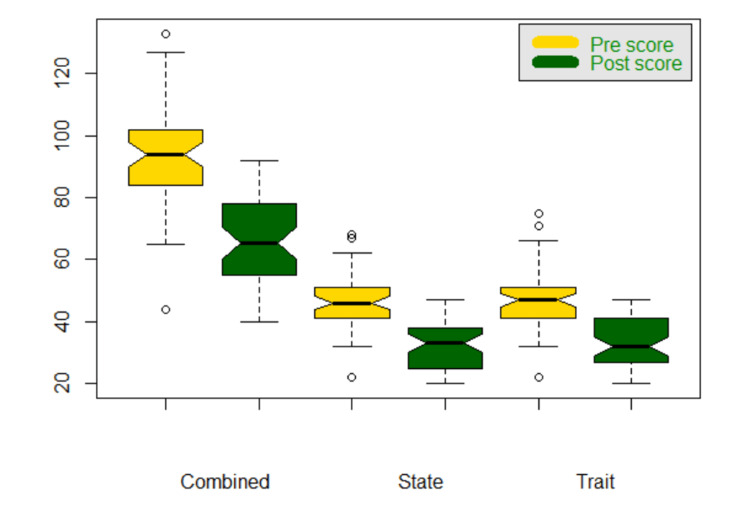
Box-plot graph of a comparison of means of pre- and post-yoga anxiety scores. Pre score - mean anxiety score on day 1 and before the intervention of yoga. Post score - mean anxiety score at the end of 6-month yogic regime.

Anxiety scores also showed a commendable drop in the values after the intervention. Mean anxiety scores on day 1, day 30, and at the end of 6 months have been depicted. This difference was statistically significant (p < 0.001) as is clear from the Table 4 and its graphical representation is depicted as a box-plot graph in Figure 2.

**Table 4:** Comparison of mean of pre- and post-yoga anxiety scores.

	Mean anxiety score		Difference in scores between day 1 & day 30 (with 95% confidence interval)	p-value	Mean anxiety score		Difference in scores between Day 1 and at the end of 6 months (with 95% confidence interval)	p-value
	Pre-yoga (on day 1)	Post-yoga (on day 30)			Pre-yoga (on day 1)	Post-yoga (at the end of 6^th^ month)		
State anxiety scores	46.82± 8.95	32.54± 7.70	14.3 (11.4-17.1)	p<0.001	46.8 ± 9.0	29.4 + 6.6	17.4 (14.7 - 20.2)	p<0.001
Trait anxiety scores	47.02± 10.70	33.06± 8.29	13.9 (11.2-16.7)	p<0.001	46.96 ± 10.7	29.9 + 7.5	17.1 (14.5 - 19.8)	p<0.001
Total anxiety scores	93.8 ± 17.73	65.62 ± 15.26	28.2 (23.1-33.4)	p<0.001	93.78 ± 17.7	59.3 + 12.9	34.6 (29.8 - 39.3)	p<0.001

To give the study more credibility and authenticity from the subject's viewpoint, participants were made to fill out a feedback questionnaire. The results were extrapolated in percentages. Table 5 gives a glance through the questionnaire. 

**Table 5:** Feedback score for various parameters expressed as a percentage of participants.

Parameters	Highly positive change	Moderately positive change	No change	Moderate negative change	Highly negative change	Consensus
Sense of contentment & well being	52	36	12	0	0	0.75
A feeling of calmness & relaxation	66	32	2	0	0	0.81
Level of concentration in studies	54	38	8	0	0	0.77
Hours required to rejuvenate	0	46	48	4	2	0.77
Self-confidence	34	44	22	0	0	0.75
Competence in any task	42	50	8	0	0	0.78
Irritability levels	42	42	16	0	0	0.75
Stamina	42	50	14	0	0	0.76
Lethargy	18	60	18	2	2	0.77
Appetite	6	22	70	2	0	0.80
Optimistic outlook in life	34	46	20	0	0	0.76
Headache, body ache	34	48	14	2	2	0.73
Mutual Interpersonal relationship	52	30	18	0	0	0.72

Apart from the improvement observed in mental well-being score, the students also reported other beneficial effects of yoga in their anonymous feedback such as:

1. Better sleep

2. Better concentration in studies

3. Better control of anger and other negative symptoms

4. More relaxed and active throughout the day

5. Getting positive energy at the beginning of the day.

## Discussion

A study incorporating the estimation of VO2 max along with psychological assessment was long due for youngsters. Once evaluated in conjunction, this could prove helpful for the pupils to get an idea of their aerobic capacity so as to modulate the intensity of different yoga practices according to their needs. This interventional study analyzed the dynamics of the cardiorespiratory responses in medical students and it was revealed that the yoga group had statistically significant higher VO₂ max and improved METS values when the fitness metrics were calculated pre- and post-yoga intervention.

Cardiopulmonary fitness assessed as VO2 max, is regarded as a critical marker for youth health. Elevated VO2 max level in yoga performers is eventually linked to better physical fitness and was a potential outcome of regular yogic practice. This finding of our study is in accordance with the studies performed by Loganathan et al. [20] and Parikh et.al. [21]. Due to a consistent yoga practice, respondents' initial sky-high anxiety levels dramatically decreased, which contributed to the inculcation of a judicious insight in young adults. Despite the fact that the youngsters who were selected for the research were healthy, their reduced aerobic capacity seemed nerve-wracking to call for a solution. When introduced to them at this juncture, yoga curbed this issue through an extensive regime tailored to their need and aimed at improving their aerobic fitness and mitigating anxiety.

Our results are consistent with previous studies [22-25] which examined the effects of yoga on the health of medical students. The paucity of data on the impact of yoga on young medical undergraduates' functional aerobic capacity during treadmill/ergometer exercise in the literature available so it was thought pertinent to utilize such a methodology, which became the study's unique selling feature. 

Additionally, the yoga group reported plausible improvements in parameters like an improved sense of well-being, a feeling of relaxation, enhanced concentration, self-confidence, better efficiency, sound interpersonal relationships, augmented attentiveness, reduced irritability levels, and an upbeat outlook on life [26-27]. A study by Bansal et al. reported significant improvement in general and mental well-being following the intervention which again corroborates our findings [28]. 

Akin to any research, this study too has certain limitations. We have used only a single composite questionnaire-based measure of anxiety and have not studied psychological factors such as appraisal and coping mechanisms that may influence the stress response. The stress scores were obtained at one point of time while they were in medical school and hence the status of the mental health of students prior to their entry to the medical course could have influenced the levels of stress. Other sources of stress such as familial or interpersonal problems were not examined. Biochemical parameters of stress such as plasma or salivary cortisol were not measured.

Nutrition was also one such factor that was not taken into sight, considering the fact that all students had the same source of food and kitchen being a part of the medical school. Hence that did not affect the readings drastically. Moreover, as a future prospect, it confers an avenue for further studies that can be undertaken in which a nutritive intervention could be used.

## Conclusions

To meet the modern lifestyle full of challenges and tensions, it has become imperative especially for medical students to bail out of this turmoil and emerge with a whole new persona. There was a significant improvement in the VO2 max and a markedly discernible decrease in anxiety levels of yoga performers. To spell out the crux of the current research, it can be conjectured that yoga can not only expound aerobic fitness but can concurrently serve to be beneficent in achieving a tranquil state of mind during young age, yet providing the concentration and arousal essential in this demanding or stressful vivency of youth.
